# Oxidative stress markers and atherogenic lipid alterations among chronic smokers

**DOI:** 10.6026/973206300214917

**Published:** 2025-12-15

**Authors:** Gaurang Hasmukhbhai Suhagiya, Soham V Shah, Krupa Rajeshbhai Gondaliya, Aditi Singh, Gazal Mangukiya, Huma Saiyad

**Affiliations:** 1 Department of Pediatrics, Shantabaa Medical College and General Hospital, Amreli, Gujarat, India; 2 Department of Surgery, Dr N.D Desai Faculty of Medical Science and Research, Dharmsinh Desai University, Nadiad, Gujarat, India; 3 Department of Casualty, Government Medical College, Surat, Gujarat, India; 4 Department of Critical Care, Dr. Baba Saheb Ambedkar Hospital, New Delhi, India; 5 Department of Emergency Medicine, B.J Medical College, Ahmedabad, Gujarat, India; 6 Department of Medicine, B.J Medical College, Ahmedabad, Gujarat, India

**Keywords:** Oxidative stress, cigarette smoking, malondialdehyde, antioxidants, cardiovascular risk, atherogenic index

## Abstract

The constant entry of exogenous reactive oxygen species makes cigarette smoking one of the key contributors to systemic oxidative stress. Therefore, it is interest to compare the oxidative stress markers and lipid profiles of chronic smokers and age matched non-smokers. A cross-sectional examination of 200 males showed that serum Malondialdehyde (MDA) level was much higher and the SOD and CAT activities were much lower among smokers. Dyslipidemia and higher Atherogenic Index of Plasma were strongly positively related to oxidative stress. Thus, those oxidative biomarkers are potential cardiovascular risk predictors in chronic smokers.

## Background:

The use of tobacco is generally declared to be a leading high profile health issue in which it is a significant contributor to the non-communicable disease burden in the world. The epidemiological evidence suggests that smoking of cigarettes can be discussed as the first risk factor to develop atherosclerosis, coronary heart disease (CHD) as well as chronic obstructive pulmonary disease (COPD) [1]. Although the addictive nature of nicotine is not an unknown phenomenon, the biochemical pathophysiology that causes the cardiovascular damage is complicated and multifactorial. The key to this disease process is the idea of oxidative stress a disruption of the equilibrium between the formation of reactive oxygen species (ROS) and the capacity of the biological system to eliminate these reactive intermediates [2]. Cigarette smoke is a complicated aerosol, which consists of more than 4700 chemical compounds, free radicals in high concentrations (superoxide anion, O 2 ( - ) Hydroxyl radical, OH ( - ) Hydrogen peroxide, H 2 O 2). It is estimated that one puff of cigarette smoke holds some 10 14 free radicals in the tar and 10 15 in the gas stages [3]. These are exogenous oxidants that cause damage to the cells by reacting with lipids, proteins and DNA. One of the main results of the interaction is lipid peroxidation, a process of chain reaction, which leads to degradation of polyunsaturated fatty acids in cell membranes, to the formation of Malondialdehyde (MDA). MDA is a biomarker that is a stable toxic aldehyde and a highly accepted measure of the amount of oxidative stress *in vivo* [4]. Human body has an enzymatic antioxidant defense system, which is mainly composed of Superoxide Dismutase (SOD) and Catalase (CAT) to fight off oxidative insults. SOD enhances the process of dismutation of the superoxide radicals into the oxygen and hydrogen peroxide, whereas CAT takes part in breaking down hydrogen peroxide into the water and oxygen. The persistent influx of ROS is believed to deplete these enzymatic reserves in chronic smokers resulting in a depleted antioxidant capacity [5]. Lipid metabolism is also inherently associated with the connection between oxidative stress and cardiovascular risk. Oxidative alteration of Low-Density lipoprotein (ox-LDL) is an essential triggering action in atherogenesis. Ox-LDL is simply chemotactic to monocytes and is easily consumed by macrophages to create foam cells, the characteristic of the initial atherosclerotic plaque [6]. Moreover, the smoking behavior has been known to change the lipid profile, popularly known as dyslipidemia of smoking, which is an increasing level of triglycerides (TG) and decreasing levels of High-Density lipoprotein (HDL). Atherogenic Index of Plasma (AIP), which is the logarithm of TG/HDL ratio, has become a sensitive predictor of cardiovascular risk, and sometimes it is better than each lipid parameter independently [7]. Therefore, it is of interest to evaluate the results of oxidative stress markers (MDA, SOD, CAT) in chronic smokers and non-smokers and to study how the selected markers are correlated with the Atherogenic Index of Plasma (AIP) to conclude about the cardiovascular risk related to the oxidative imbalance.

## Materials and Methods:

## Study design and setting:

This cross-sectional comparative study was conducted at the Department of Biochemistry and the General Medicine Outpatient Department of a tertiary care university hospital. The study duration was six months, from January 2023 to June 2023.

## Study population:

The sample size was calculated based on a power of 80% and a confidence level of 95%, resulting in a total of 200 male participants aged between 25 and 50 years. The participants were divided into two groups:

[1] Group I (Smokers): 100 subjects who had smoked at least 10 cigarettes per day for a duration of more than 5 years.

[2] Group II (Non-Smokers): 100 age- and Body Mass Index (BMI)-matched healthy subjects who had never smoked.

## Inclusion and Exclusion Criteria:

Inclusion criteria for the study group included active smoking status and absence of acute illness. Exclusion criteria for both groups were: history of diabetes mellitus, hypertension, renal or hepatic failure, thyroid dysfunction, current use of lipid-lowering drugs, alcohol dependence, and intake of vitamin supplements (Vitamin C or E) within the last month, as these could confound oxidative stress measurements.

## Data collection and sampling:

Demographic data, including age, BMI, and smoking history (calculated as pack-years), were obtained via a structured questionnaire. Blood pressure was measured using a standard mercury sphygmomanometer. Under aseptic conditions, 5 mL of venous blood was collected from the antecubital vein of each participant after an overnight fast of 10-12 hours. The blood was allowed to clot for 30 minutes at room temperature and then centrifuged at 3000 rpm for 10 minutes to separate the serum. The serum was aliquoted and stored at -80°C until analysis.

## Biochemical analysis:

[1] Estimation of MDA: Serum Malondialdehyde levels were estimated using the Thiobarbituric Acid Reactive Substances (TBARS) assay. MDA reacts with thiobarbituric acid to form a pink chromogen, the absorbance of which was measured spectrophotometrically at 532 nm.

[2] Estimation of SOD: Superoxide Dismutase activity was assayed based on the inhibition of the auto-oxidation of pyrogallol at pH 8.0. The absorbance was read at 420 nm.

[3] Estimation of CAT: Catalase activity was measured by following the decomposition of hydrogen peroxide (H_2 O_2). The decrease in absorbance was monitored at 240 nm.

[4] Lipid Profile: Serum Total Cholesterol (TC), Triglycerides (TG), and High-Density Lipoprotein (HDL) were analyzed using standard enzymatic colorimetric kits on an automated chemistry analyzer. Low-Density Lipoprotein (LDL) was calculated using the Friedewald formula: LDL=TC-(HDL+TG/5).

## Cardiovascular risk assessment:

The Atherogenic Index of Plasma (AIP) was calculated using the formula:

AIP=log⁡(Triglycerides/HDL"-" Cholesterol)

An AIP value of < 0.11 is considered low risk, 0.11-0.21 is intermediate risk, and > 0.21 is considered high cardiovascular risk.

## Statistical analysis:

Data were analyzed using SPSS software version 25.0. Results were presented as Mean ± Standard Deviation (SD) or percentages. The significance of differences between groups was assessed using the Independent Student's t-test. Pearson's correlation coefficient (r) was used to analyze the relationship between oxidative stress markers and lipid parameters. A p-value of < 0.05 was considered statistically significant.

## Results:

The study included 200 participants, with 100 in the smoker group and 100 in the non-smoker control group. The demographic and baseline clinical characteristics are summarized in Table 1 (see PDF). There were no statistically significant differences between the two groups regarding age and BMI (p>0.05), ensuring that the groups were well-matched. However, systolic and diastolic blood pressures were significantly higher in the smoker group (p<0.05).

## Oxidative stress markers:

The analysis of oxidative stress markers revealed a distinct imbalance in the smoker group (Table 2 - see PDF). Serum MDA levels, indicating lipid peroxidation, were significantly elevated in smokers (5.82±1.14 µmol/L) compared to non-smokers (2.15±0.68 µmol/L; p<0.001). Conversely, the activities of antioxidant enzymes were markedly suppressed in smokers. SOD activity was significantly lower in smokers (112.4±14.2 U/mL) versus non-smokers (145.6±18.5 U/mL), and CAT activity showed a similar decline (p<0.001 for both).

## Lipid profile and cardiovascular risk:

Table 3 (see PDF) illustrates the lipid profile and cardiovascular risk indices. Smokers exhibited a dyslipidemic profile characterized by significantly higher Total Cholesterol, Triglycerides, and LDL-Cholesterol compared to non-smokers. Notably, HDL-Cholesterol (protective cholesterol) was significantly lower in the smoker group (36.2±4.5 mg/dL vs 48.5±5.2 mg/dL). Consequently, the Atherogenic Index of Plasma (AIP) was significantly higher in smokers (0.41±0.12) compared to non-smokers (0.12±0.08), placing the average smoker in the "high cardiovascular risk" category. Pearson correlation analysis (data not tabulated) indicated a strong positive correlation between MDA and AIP (r=0.68,p<0.01) and a negative correlation between SOD and AIP (r=-0.55,p<0.01) in the smoker group.

## Discussion:

The current research offers strong evidence on the harmful effects of prolonged smoking of cigarettes on the oxidative balance and cardiovascular conditions. This minimized confounding variables by pairing the participants on age and BMI, and, therefore, enabled easier attribution of the observed metabolic changes to smoking status. We have shown that there is almost a tripled Malondialdehyde (MDA) level in the serum of smokers, when compared to non-smokers. This is in keeping with the process whereby cigarette smoke, a source of exogenous free radicals, triggers the process of peroxidation of polyunsaturated fatty acids of cell membranes. The increase of MDA is a critical observation since MDA is not an active damage marker but rather is an active mutagenic and atherogenic molecule. It is able to cross-link proteins and alter LDL particles and make them immunogenic. At the same time, there was a marked decrease in the SOD and CAT activity among the smokers. This is a twofold reduction in terms of its biological plausibility. To begin with, the enzymes are utilized more intensively to counter the huge amount of exogenous ROS produced by cigarette smoking. Second, tobacco smoke components including cadmium and nicotine can be able to directly suppress the biosynthesis or active sites of these enzymes. Howsoever, Rahman and MacNee have already explained how cigarette smoke brings about an oxidant/antioxidant imbalance in the lung and systemic circulation whereby the antioxidant screen of the epithelial lining fluid is overpowered [9]. The important negative relationship which is found between the level of antioxidants and the year of smoking (pack-years) in related studies also supports the view that this is dose-dependent cumulative depletion [10]. With the lipid profile analysis, the smokers were found to have a typical atherogenic pattern of high TG and LDL and low HDL. Insulin resistance and inhibition of lipoprotein lipase (LPL) activity are the mechanism that connects smoking and dyslipidemia. The nucleus acts by increasing release of catecholamines, thus causing lipolysis in adipose tissues releasing free fatty acids into circulation that get converted into triglycerides/VLDL by the liver [11]. The decreasing levels of HDL are of great concern because HDL is involved in reverse cholesterol transport, as well as antioxidant properties through enzyme paraoxonase-1 (PON1). Smoking has been demonstrated to suppress PON1 activity thus, worsening the functional quality of HDL, and not the amount [12, 13]. Another critical point in the present research was the correlation analysis of the oxidative markers and the Atherogenic Index of Plasma (AIP). The positive relationship existing between AIP and MDA is strong and positive which implies that oxidative stress does not merely co-exist with dyslipidemia but is a contributor to dyslipidemia. ROS is capable of suppressing the action of Lecithin-Cholesterol Acyltransferase (LCAT), which is involved in the maturation of HDL, which gives a molecular basis to the low levels of HDL in high-oxidative stress conditions [14]. Moreover, oxidized LDL (generated by high MDA and low SOD) is cytotoxic to endothelial cells, and it induces the release of proinflammatory cytokines and adhesive molecules, and atherosclerotic plaques are formed faster [15, 16]. In contrast to other studies which consider old age smokers, our use of a younger group (25 to 50 years) emphasizes the fact that such biochemical derangements are relatively early in the smoking habit, way before clinical findings of heart diseases can be detected. This highlights the fact that oxidative pathology is silent [17]. The research has its shortcomings due to its cross-sectional nature, which does not allow making any causal inferences. We also did not determine other antioxidants such as Glutathione or Vitamin C which would give us a clearer picture of the total antioxidant capacity. Male participants were also used to eliminate hormonal differences in the female population that may influence the metabolism of lipid which limited the generalization of results to the female population of smokers. It is advised to conduct future longitudinal studies that will monitor these markers after the smoking cessation [18].

## Conclusion:

Chronic cigarette smoking is associated with a pronounced imbalance between oxidative stress and antioxidant defense, evidenced by elevated malondialdehyde levels and reduced superoxide dismutase and catalase activities. This oxidative disturbance is closely linked to an atherogenic lipid profile and a significantly increased Atherogenic Index of Plasma. Collectively, these findings support the role of oxidative stress-mediated metabolic alterations as an early biochemical pathway contributing to increased cardiovascular risk in chronic smokers.
